# Clinical and functional results after implantation of the bonebridge, a semi-implantable, active transcutaneous bone conduction device, in children and adults

**DOI:** 10.1007/s00405-021-06626-7

**Published:** 2021-03-06

**Authors:** Ingmar Seiwerth, Laura Fröhlich, Sebastian Schilde, Gerrit Götze, Stefan K. Plontke, Torsten Rahne

**Affiliations:** 1 Department of Otorhinolaryngology, Head & Neck Surgery, Martin Luther University Halle-Wittenberg, University Medicine Halle (Saale), Ernst-Grube-Str. 40, 06120 Halle, Germany; 2 Department of Orthopedics, Trauma and Reconstructive Surgery, Martin Luther University Halle-Wittenberg, University Medicine Halle (Saale), Halle, Germany

**Keywords:** Transcutaneous hearing implant, Bone-anchored hearing aid, Bone conduction implant, Conductive hearing loss, Mixed hearing loss

## Abstract

**Purpose:**

Aim of the study was to evaluate the surgical, clinical and audiological outcome of 32 implantations of the Bonebridge, a semi-implantable transcutaneous active bone conduction implant.

**Methods:**

In a retrospective cohort study, we analyzed data for 32 implantations in 31 patients (one bilateral case; seven age < 16 years) with conductive or mixed hearing loss, malformations, after multiple ear surgery, or with single-sided deafness as contralateral routing of signal (CROS).

**Results:**

Four implantations were done as CROS. Five cases were simultaneously planned with ear prosthesis anchors, and 23 implantations (72%) were planned through three-dimensional (3D) “virtual surgery.” In all 3D-planned cases, the implant could be placed as expected. For implant-related complications, rates were 12.5% for minor and 3.1% for major complications. Implantation significantly improved mean sound field thresholds from a preoperative 60 dB HL (SD 12) to 33 dB HL (SD 6) at 3 postoperative months and 34 dB HL (SD 6) at > 11 postoperative months (*p* < 0.0001). Word recognition score in quiet at 65 dB SPL improved from 11% (SD 20) preoperatively to 74% (SD 19) at 3 months and 83% (SD 15) at > 11 months (*p* < 0.0001). The speech reception threshold in noise improved from − 1.01 dB unaided to − 2.69 dB best-aided (*p* = 0.0018).

**Conclusion:**

We found a clinically relevant audiological benefit with Bonebridge. To overcome anatomical challenges, we recommend preoperative 3D planning in small and hypoplastic mastoids, children, ear malformation, and simultaneous implantation of ear prosthesis anchors and after multiple ear surgery.

## Introduction

In 2012, the Bonebridge (BCI 601) was introduced by MED-EL, Innsbruck, Austria. This semi-implantable, active, transcutaneous bone conduction (BC) hearing device came into widespread use in the treatment of conductive and mixed hearing loss or in single-sided deafness (SSD). Two years after it debuted, more than 200 centers worldwide had used it [1].

The implantable part of the Bonebridge consists of the floating mass transducer (FMT), the demodulator, and a coil for receiving data from the sound processor, which is held outside on the skin surface by a magnet in the receiver coil. Thus, no skin penetration is necessary for sound transmission, representing a major advantage compared to percutaneous active BC devices in use for the same indication for more than 30 years [2]. Skin penetration has been associated with higher risks of wound infections and complications [3]. Speech recognition and sound field threshold outcomes of the Bonebridge and bone-anchored hearing solutions can be considered equivalent [4].

The designated site for implantation of the FMT is the mastoid (sinodural angle or retrosigmoidal). Placement in the squamous portion of the temporal bone also has been described [5]. The FMT is fixed in the temporal bone with two screws that do not require osseointegration and that transmit the vibrations from the FMT to the bone. A detailed description of the surgical procedure has been published previously [1, 6, 7].

Following the manufacturer’s guidelines, the Bonebridge is indicated for the following: conductive or mixed hearing loss with BC thresholds ≤ 45 dB HL (4PTA_BC_); as a contralateral routing device in cases of SSD; with insufficient hearing rehabilitation after (multiple) tympanoplasty; after canal wall down mastoidectomy; in otitis media and externa, when conventional hearing rehabilitation is not possible; and in malformations [1, 8]. In the European Union, the device is CE-approved for patients age 5 years and older.

Although this implant offers obvious advantages as an active BC implant without skin penetration, the dimensions of the cylindrical FMT (8.7 mm depth, 15.8 mm diameter) can be a limitation regarding its surgical applicability. For this reason, careful preoperative planning based on computed tomography (CT) scan data of the temporal bone is required for evaluation of implantability and to avoid a relevant impression of intracranial soft tissue structures such as the dura mater and sigmoid sinus.

The aim of this study was to evaluate the surgical, clinical and functional outcome for patients who received the device in our implant center from June 2012 until May 2019.

## Methods

### Patients

We retrospectively analyzed the data for all cases involving Bonebridge implantations (BCI 601, MED-EL, Innsbruck, Austria) in our department, a tertiary referral center, from June 2012 to May 2019. Our analysis included medical history, indication, preoperative planning, surgery, and short- and long-term clinical and audiological outcomes. The study was conducted with the approval of the local ethics committee (No. 2019–123) and in accordance with the Declaration of Helsinki.

### Surgery and Preoperative Planning

**Fig. 1:**
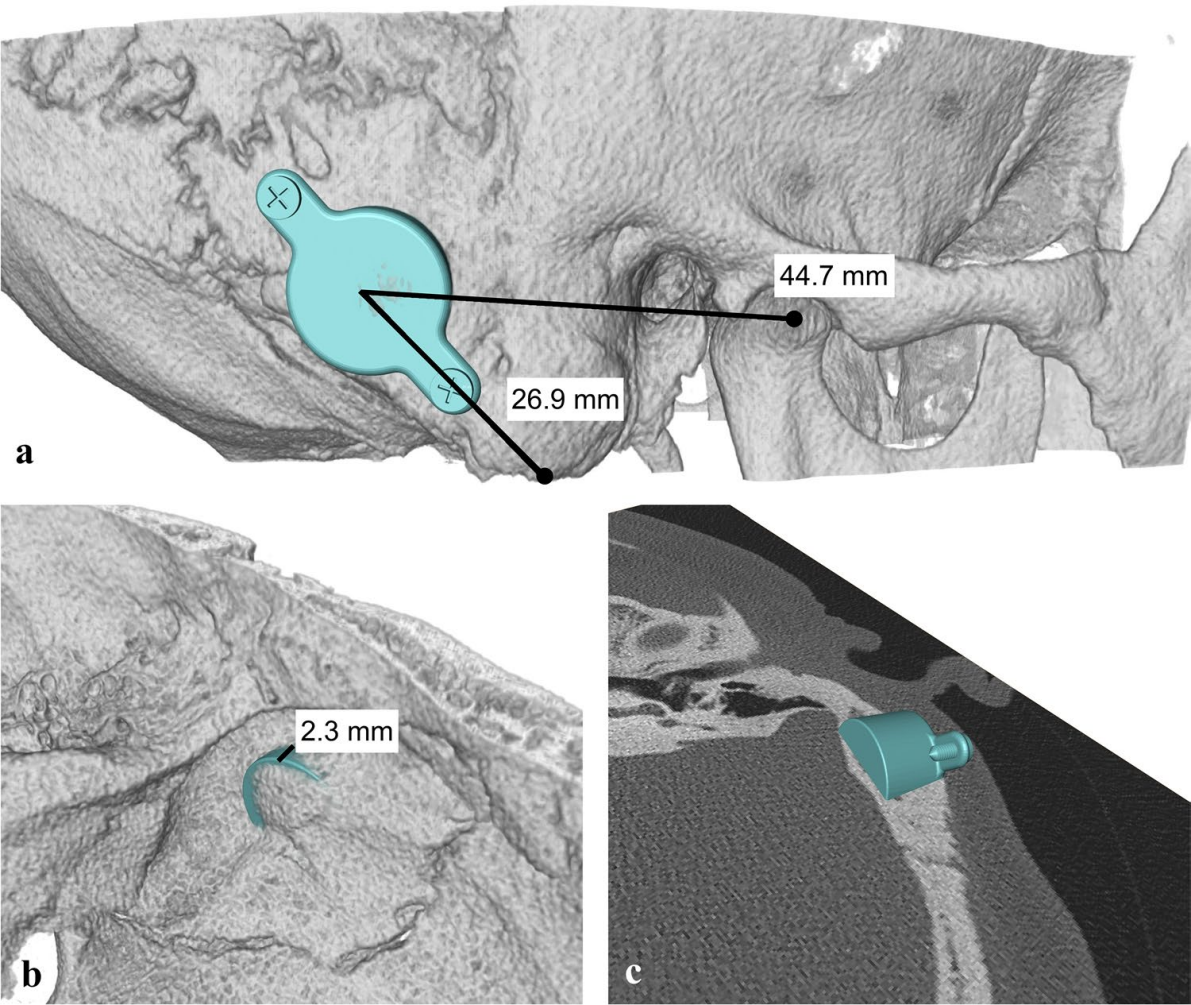
Example of optimal FMT position determined by preoperative 3D planning (“virtual surgery”). **a** Extracranial view with distances to landmarks for intraoperative transfer. **b** Intracranial view, where a partial impression of the sigmoid sinus indicates the necessity of the use of lifts. **c** Verification of the implant position overlying the axial CT scan layer

All implantations were carried out by the same surgeon (SKP) as an in-patient-procedure under general anesthesia. For determining the optimal implant position, preoperative planning was performed as “virtual surgery” with Amira software (FEI Visualization Sciences Group, Burlington, USA) using three-dimensional (3D) reconstructed models of the implant and the temporal bone based on CT scan data (Fig. 1). Referring to anatomical landmarks, the designated FMT position was translated to the intraoperative situation. A detailed description of the 3D planning method was reported elsewhere [6]. Surgery was conducted as instructed in the manufacturer’s handbook respecting standard principles of temporal bone/middle ear surgery.

After an adequate healing period, an Amadé, or, after its market introduction, a Samba audio processor was applied and fitted using Symfit within Connexx software (Sivantos GmbH under Trademark License of Siemens AG, Erlangen, Germany).

### Audiological Assessment

Pure-tone thresholds were measured for air conduction (AC) and BC before surgery and at 1–3 months and > 11 months (long term) after surgery. Postoperatively, aided thresholds were measured using warble tones. Speech recognition for monosyllabic words and multisyllabic numbers in quiet was measured using the German Freiburg or Mainz speech perception tests at 65 dB SPL (WRS_65_). Before surgery, we measured speech recognition for the unaided and best-aided conditions, and we made these measurements after surgery using the respective audio processor. Speech recognition in noise was measured with the German Oldenburg matrix test (HörTech GmbH, Oldenburg, Germany) for adults or children. The 50% speech reception threshold in a constant noise level of 65 dB SPL (SRT_noise_) was measured postoperatively with speech and noise presented from the front (S_0_N_0_) in aided and unaided conditions.

In situ pure-tone thresholds were measured following the manufacturer’s vibrogram procedure. In this measurement mode, the external audio processor drives the FMT based on defined amplification parameters, and the outcome is measured as behavioral thresholds, according to pure-tone audiometry. Because vibrogram measurements require the use of the most recent model of an audio processor, the number of participants for this test was only 19. The four individuals implanted as contralateral routing of signal (CROS) were excluded from further common audiological analyses except for preoperative and postoperative BC measurements. For these patients, preoperative BC thresholds of the contralateral ear are additionally indicated in Table 2.

Pure-tone thresholds of BC (4PTA_BC_) and AC (4PTA_AC_) and vibrogram results are presented as the mean (standard deviation; SD) value of the data measured at 0.5, 1, 2, and 4 kHz.

### Statistical analysis

Statistical analysis was conducted in pairwise comparisons with paired t-tests or with Wilcoxon matched-pairs signed-rank tests if data were not normally distributed. For comparison of more than two time points, we applied repeated-measured analysis of variance with Tukey’s multiple comparison tests or a mixed-effects analysis with Holm–Sidak’s multiple comparisons test if values were missing at some time points. The level of significance was set at *p* < 0.05. Statistical analysis was calculated and graphics designed using Prism 7 software (GraphPad, La Jolla, CA, USA).

## Results

### Demographics

Between July 2012 and May 2019, a total of 32 Bonebridge implantations were carried out in 31 patients (one patient had bilateral implantation in different sessions). Table 1 gives a detailed overview of demographics and clinical data. The mean age at implantation was 38 years (SD 22; range, 5–74 years). Seven patients were children and adolescents younger than 16 years old; 23 patients were male, and 8 patients were female.

**Table 1:** Demographic and clinical data of 32 implantations

Surgery ID	Age at surgery (years)	M/F	Treated side	BC-FMT position	Diagnosis/indication	Otologic history (treated ear)	Additional surgery
1	10	M	R	SDA	Malformation (Altmann Type II, microtia, EAC atresia)	Softband-BAHA, BAHA dismissed	None
2	49	M	L	SDA	Bilat. ear malformation and ear canal atresia, chronic secreting OE	Ear canal surgery and CWD surgery in childhood	simultaneous implantation of epithesis anchors
3	37	M	R	SDA	Malformation of ear canal and external ear	None	None
4	59	F	R	SDA	CHA not tolerated	Surgery for chole and TPL III (PORP)	Granulation tissue removal in antrum and epitympanum
5	50	F	L	SDA	ME chole, post-inflammatory stapes fixation	TPL I, surgery for chole, TPL rev. I	Chole surgeryand TPL rev. III (PORP)
6	37	M	R	SDA	Recurrent OE, recurrent chole in open mastoid cavity; CHA not successful	CWD (chole); TPL rev., partial cavity obliteration	Cavity rev. and partial obliteration, cartilage and Palva flap, TPL III (PORP); removal of a Tornwaldt cyst
7	9	M	L	SDA	Malformation (Altmann type II), craniofacial dysmorphy	Bifrontal craniotomy and bifrontal advancement (metopic synostosis)	None
8	13	M	L	SDA	Recurrent OE, CHA not successful	TPL I; TPL rev. I; surgery for chole, TPL-rev. III (PORP); TPL-rev. III (PORP)	Tympanoscopy, exclusion of residual chole, TPL-rev. III (PORP)
9	53	M	R	SDA	Radical cavity, post-inflammatory fibrosis, CHA not tolerated	2 ear cavity surgeries in childhood; CWD chole surgery, dura enforcement tegmen mastoideum, occlusion labyrinthine fistula III° of the basal cochlea, TPL III (facial nerve exposed)	None
10	35	F	R	SDA	SSDCROS	Vestibular schwannoma surgery retrosigmoidal	None
11	30	M	R	SDA	Post-inflammatory stapes footplate fixation and ossification of RW niche, AMEI intra-OP not applicable	TPL, TPl III rev. (chole, TORP), 2. TPL-rev. and antrotomy	Tympanoscopy
12	76	M	L	SDA	Secreting open mastoid cavity and ME fibrosis left; CHA not tolerated; bone conduction hearing glasses unsatisfying	CWD surgery (chole)	Cavity rev. and partial obliteration, TPL III rev. (TORP)
13	52	M	L	SDA	Secreting COMM, inflamed radical cavity	CWD surgery, cavity-rev., TPL III (TORP)	Tissue excision in mastoid cavity
14	46	F	R	SDA	Secreting radical cavity, CHA not successful	CWD surgery	Granulation tissue removal from cavity
15	46	M	L	SDA	Secreting and granulating COMM and infected radical cavity, CHA not successful	3 ear surgeries (TPL and radical cavity)	Cavity rev., cavity reduction, ear canal widening; balloon dilatation of the eustachian tube
16	54	F	R	SDA	SSDCROS	CROS with CHA not tolerated, CI dismissed	None
17	7	M	R	SDA	Malformation (microtia, EAC atresia, incus and malleus dysplasia)	Further use of softband-BAHA and BAHA dismissed	None
18	6	M	R	SDA	Malformation(Altmann Type II, microtia, EAC atresia, dysplasia of malleus and incus, pathological facial nerve location)	None	Tympanoscopy, AMEI not possible
19	49	M	R	SDA	Bilat. ear malformation and ear canal atresia, chronic secreting OE	Ear canal surgery and CWD surgery in childhood	Resection of ear rudiments, simultaneous implantation of epithesis anchors
20	12	M	L	SDA	Bilat. malformation with bony obstruction of oval window, stapes malformation and pathologic facial nerve location	Tympanoscopy	
21	51	M	R	SDA	Chronic OE, partial tympanic fibrosis, post-inflammatory stapes fixation, chronic mastoiditis	CWD surgery	Resection of Concha rudiments, simultaneous implantations of epithesis anchors for ear epithesis
22	17	F	R	SDA	Chronic secreting myringitis and OE, COMM and tympanic fibrosis, CHA insufficient	TPL I; 2 TPL rev.	TPL III rev. (PORP), ear canal rev.
23	5	M	R	SDA	Malformation (microtia, ear canal stenosis)	Further use of softband-BAHA and BAHA dismissed	None
24	61	M	R	SDA	OE exacerbation with HA; secreting COMM and OE; ME fibrosis right	2 TPLs	TPL III rev. (PORP), ear canal surgery split thickness skin grafts;
25	42	M	L	SDA	SSD, chole, sclerosis of cochlea and labyrinthCROS	None	Chole surgery, canal wall reconstruction
26	5	M	L	SDA	ME malformation (stapes footplate agenesis, narrow and flat oval window niche, dysplasia of the superstructure of the stapes, hypoplasia of the long incus process); CHA and softband-BAHA deselection	Tympanoscopy, atticotomy	None
27	65	M	L	SDA	Near deafness, chronic secreting OE with post-inflammatory fibrosis; benefit of CROS-BAHA softband testing; CI not possibleCROS	Ear canal surgery, tympanoscopy; ear canal widening rev., split thickness skin grafts, adhesiolysis ME, TPL I	None
28	73	M	R	SDA	Severe chronic OE, partial tympanic fibrosis, post-inflammatory stapes fixation, chronic mastoiditis	Ear canal exostoses surgery, ear canal widening, TPL III (PORP)	Mastoidectomy, ear canal- rev., TPL-rev., occlusion of perilymphatic fistula RW-niche (AMEI not possible)
29	45	M	R	SDA	Ear malformation	2 TPL-rev.; implantation and re-implantation of epithesis anchors	None; epithesis anchors already in situ
30	17	F	L	RS	Malformation (microtia, EAC atresia, incus and malleus dysplasia)	Multiple failed reconstructions of the external ear	Simultaneous implantations of anchors for ear epithesis
31	71	F	L	RS	Inflamed secreting open cavity with widely exposed dura, partial bone necrosis	Chole surgery; chole-rev., occlusion of liquor fistula, TPL III	Cavity rev., lateral petrosectomy, dura enforcement, occlusion of the tympanic opening of the eustachian tube, occlusion of external ear canal, cavity obliteration with abdominal fat
32	26	M	L	SDA	Chronic secreting OE left	CWD surgery (chole) and labyrinthine fistula LSCC; partial cavity obliteration and TPL III (PORP), 2nd look and TPL-rev. III (PORP)	Cavity rev. and split thickness skin grafts left

*AMEI* active middle ear implant, *BC-FMT* bone conduction floating mass transducer; *BAHA* bone-anchored hearing aid, *CHA* conventional hearing aid, *Chole* cholesteatoma, *CI* cochlear implant, *COMM* chronic mesotympanic otitis media, *CROS* contralateral routing of signal, *CWD* canal wall down mastoidectomy, *EAC* external auditory canal, *F* female, *ID* identification number, *L* left, *LSCC* lateral semicircular canal, *LTFU* lost to follow-up, *M* male, *ME* middle ear, *OE* otitis externa, *PORP* titanium partial ossicular reconstruction prosthesis, *R* right, *RS* retrosigmoidal, *rev.* revision surgery, *RW* round window, *SDA* sinodural angle, *SSD* single-sided deafness, *TPL* tympanoplasty type I or III, *TORP* titanium total ossicular reconstruction prosthesis

### Indications and Surgery

Implantation was conducted based on insufficient hearing rehabilitation after (multiple) tympanoplasty and secreting radical cavity (n = 8), medical indication because of recurrent chronic otitis externa when using hearing aids and not solvable by canaloplasty (n = 8), malformations (n = 12), or in SSD as CROS (n = 4). In four patients, five implantations of the Bonebridge were planned considering a simultaneous implantation of anchors for ear prosthesis. In one of these patients, implantation was performed on both sides in two different sessions, whereas in one patient, prosthesis anchors were already in situ. In 30 implantations, the sinodural angle served as the implantation site, and in two cases, a retrosigmoidal placement was necessary. The 3D preoperative planning was conducted as virtual surgery in 23 implantations (72%), whereas nine surgeries (28%) were planned conventionally (two dimensionally) with standard temporal bone CT scans.

When drilling the FMT bed, the dura or the sigmoid sinus was not exposed or impressed in 10 cases (31%). In the other 10 cases (31%), an impression of the dura or sigmoid sinus of ≤ 2 mm was necessary. All other implantations were completed with a partial exposure or by skeletonizing the sinus or the dura, but without impression. The use of BCI lifts was necessary in 10 implantations (31%). In all cases with preoperative 3D planning, the implant could be placed as indicated in the virtual surgery, as assessed by the visual exploration of the surgical situs.

### Audiological Results

**Fig. 2:**
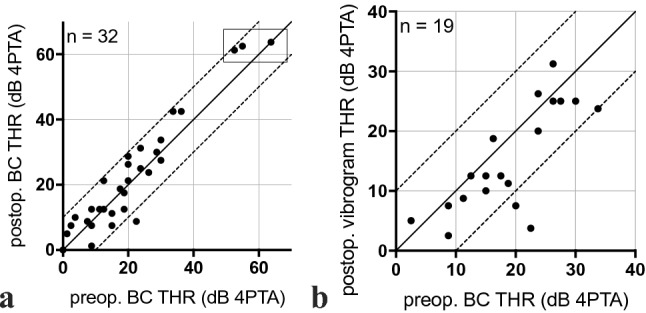
Safety. **a** Scatterplot of pre- and postoperative BC thresholds (THR, 4PTA). CROS patients are indicated within the frame (*n* = 4; 2 values overlap). **b** Scatterplot of preoperative BC thresholds and postoperative vibrogram thresholds (4PTA) showing sufficient transfer of vibrational energy to the inner ear and an adequate implant function

Individual audiological outcome for the patients is reported in Table 2. Safety regarding inner ear hearing preservation was evaluated based on the comparison of pre- and postoperative BC measurement (4PTABC). Sufficient transfer of vibrational energy to the inner ear was further evaluated by the vibrogram results in comparison to the BC thresholds. Considering an accuracy of BC threshold measurements of ± 5 dB, all individuals showed stable BC thresholds (Fig. 2a) and an adequate implant function (Fig. 2b).

**Table 2:** Audiological data

Surgery ID	Treated side	Preoperative		WRS (% at 65 dB SPL)		Postoperative					SRTnoise (dB SNR)		Vibrogram
		4PTA (dB HL)				4PTA (dB HL)			WRS (% at 65 dB SPL)				
		BC	AC	UA	BA	BC	AC 3M aided SF	AC >11M aided SF	3M aided	>11M aided	UA	BA	4PTA
1	R	0	52.5	0	80	0	25	30	90	90	− 3.6	− 5.2	0
2	L	20	62.5	5	80	28.75	45	45	60	70	3.2	− 0.5	25
3	R	18.75	NM	5	NM	17.5	41.25	30	70	LTFU	LTFU	LTFU	18.75
4	R	8.75	40	40	60	12.5	28.75	28.75	90	95	− 1.3	− 1.7	7.5
5	L	20	70	0	40	21.25	26.25	31.25	95	100	− 5.7	− 5.2	7.5
6	R	12.5	38.75	40	95	21.25	26.25	NM	95	100	− 3.8	− 3.9	n/a
7	L	2.5	47.5	0	100	7.5	37.5	33.75	80	90	NM	NM	5
8	L	3.75	38.75	70	100	10	32.5	32.5	45	90	1.3	6	0
9	R	30	75	0	45	27.5	35	41.25	60	100	NM	NM	25
10	R	R: 63.75 L: 8.75	110	N/A	N/A	> 63.75	N/A	N/A	N/A	N/A	N/A	N/A	N/A
11	R	30	67.5	0	50	33.75	35	LTFU	65	LTFU	− 3.2	− 3.4	0
12	L	28.75	76.25	0	75	30	46.25	48.75	85	75	2.9	0.7	0
13	L	18.75	51.25	25	95	12.5	28.75	28.75	85	90	− 0.5	− 3.1	11.25
14	R	11.25	38.75	65	100	12.5	27.5	30	95	100	− 5.3	− 5.2	N/A
15	L	26.25	62.5	0	80	23.75	32.5	31.25	80	90	− 3.4	− 3.5	25
16	R	R: 52.5 L: 2.5	90	N/A	N/A	61.25	N/A	N/A	N/A	N/A	N/A	N/A	N/A
17	R	7.5	60	0	70	8.75	20	NM	75	90	− 2.8	− 3.6	0
18	R	1.25	62.5	0	50	5	30	36.25	100	80	NM	NM	N/A
19	R	23.75	58.75	15	90	25	40	40	35	80	> 20	3.2	N/A
20	L	8.75	60	0	60	1.25	30	NM	75	65	NM	NM	2.5
21	R	33.75	85	0	50	42.5	41.25	33.75	55	70	− 1	− 2.4	N/A
22	R	17.5	55	0	85	18.75	27.5	30	85	60	− 4.8	− 6	12.5
23	R	22.5	77.5	0	40	8.75	25	25	90	100	− 1.6	− 4	N/A
24	R	20	61.25	0	55	26.25	38.75	42.5	45	75	2	− 1.6	0
25	L	L: 63.75 R: 20	110	N/A	N/A	>63.75	N/A	N/A	N/A	N/A	N/A	N/A	N/A
26	L	15	60	10	90	11.25	33.75	32.5	90	95	NM	NM	12.5
27	L	L: 55 R: 22.75	78.75	N/A	N/A	> 62.5	N/A	N/A	N/A	N/A	N/A	N/A	N/A
28	R	36.25	67.5	0	85	42.5	36.25	NM	40	40	NM	NM	N/A
29	R	12.5	72.5	0	60	12.5	35	35	55	80	− 2.5	− 3.8	N/A
30	L	15	67.5	0	NM	7.5	32.5	28.75	95	95	− 5.1	− 5.5	10
31	L	23.75	65	0	80	31.25	37.5	27.5	70	80	− 2.4	− 4	20
32	L	8.75	55	25	65	7.5	35	NM	55	70	− 3.5	− 3.7	N/A

*4PTA* pure-tone average 0.5; 1; 2; 4 kHz, *AC* air conduction, *BA* best aided, *BC* bone conduction, *dB* decibel, *HL* hearing level, *ID* identification number, *L* left, *LTFU* lost to follow-up, *M* months, *N/A* not available, *NM* not measured, *R* right, *SF* sound field, *SNR* signal-to-noise ratio, *SPL* sound pressure level, *SRTnoise* speech reception threshold in noise, *UA* unaided, *WRS* word recognition score

**Fig. 3:**
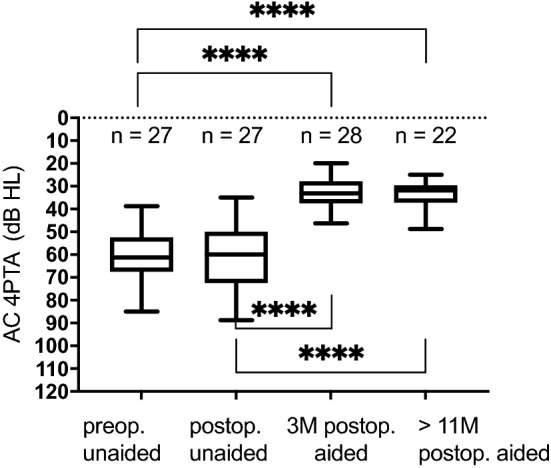
Distribution of preoperative (preop.) and postoperative (postop.) unaided and at 3 months (3 M) and > 11 months (> 11 M) postop. Aided AC thresholds (4PTA) as a boxplot. Significance is indicated with **** (*p* < 0.0001). Aided condition was measured in the sound field

Figure 3 shows the unaided and aided sound field thresholds. After implantation and audio processor fitting, the mean aided sound field thresholds improved to 33 dB HL (3 months, SD 6) and 34 dB HL (> 11 months, SD 6) compared to the preoperative unaided measures (60 dB HL, SD 12; *p* < 0.0001).

**Fig. 4:**
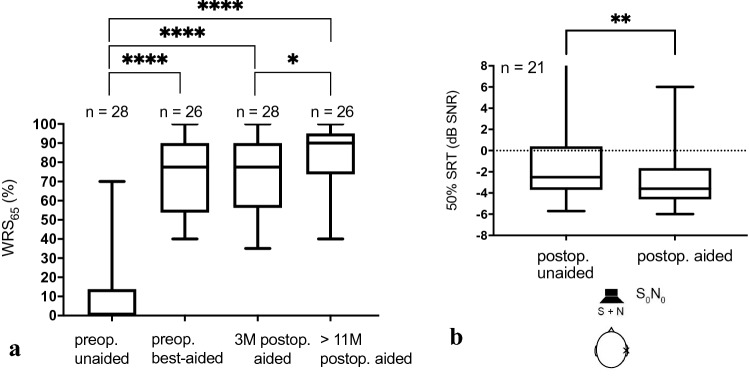
Speech recognition in quiet and in noise. **a** Distribution of preoperative (preop.) unaided, preop. best-aided, at 3 months (3 M) and > 11 months (> 11 M) postoperative (postop.) word recognition scores (WRS_65_, 4PTA) as a boxplot. Significance is indicated with **** (*p* < 0.0001) or * (*p* = 0.019). **b** Boxplot of speech recognition thresholds in noise (SRT, 50% correct in Oldenburg sentence test) with the frontal presentation of speech signal and noise (S_0_N_0_). Significance is indicated with ** (*p* = 0.0018)

Mean word recognition scores improved to 74% (SD 19) at 3 months and 83% (SD 15) at > 11 months after audio processor fitting compared to the preoperative situation (11%, SD 20; *p* < 0.0001). Long-term follow-up results indicated a significant improvement compared to 3 months after implantation (*p* = 0.019; Fig. 4a).

Speech recognition in noise also improved significantly after implantation. The mean SRT_noise_ in the S_0_N_0_ condition decreased from − 1.01 dB signal-to-noise ratio (SNR; SD 5.48) to − 2.69 dB SNR (SD 2.97; *p* = 0.0018; Fig. 4b).

### Complications

In two cases, a minor intraoperative complication occurred. In patient 4, bleeding from a dural vessel needed to be managed, and a small cerebrospinal fluid leak on the tegmen of the antrum was controlled. In this case, a partial mastoidectomy and antrotomy were performed. In the second case (patient 7), bleeding from a dural vessel needed to be controlled, as well. Patient 4 also experienced a minor complication 23 months after surgery, a reddening and swelling of the skin in the implant area, attributed to acute otitis media. Symptoms receded completely after treatment with antibiotics and paracentesis. In patient 3, a subcutaneous suture protruded from the cranial wound pole at 30 days after surgery and was easily removed during an outpatient visit.

Four complications involved revision surgery and were classified as major complications or serious adverse events. Patient 21, who had a history of two previous (unsuccessful) ear surgeries for auricular reconstruction by a different group, received prosthesis anchors simultaneously with the Bonebridge implant. This patient then experienced wound healing problems with partial skin necrosis at the anterior edge of the FMT, requiring revision surgery with a rotational flap 22 days after implantation. Patient 28 experienced attachment problems with the sound processor after surgery. At 42 days after implantation, a thinning of the scalp over the coil was necessary and conducted under local anesthesia. In patient 19, inflamed granulation tissue had to be removed from around the prosthesis anchor approximately 2 months postoperatively. However, this inflammation was not related to the implant device.

**Fig. 5:**
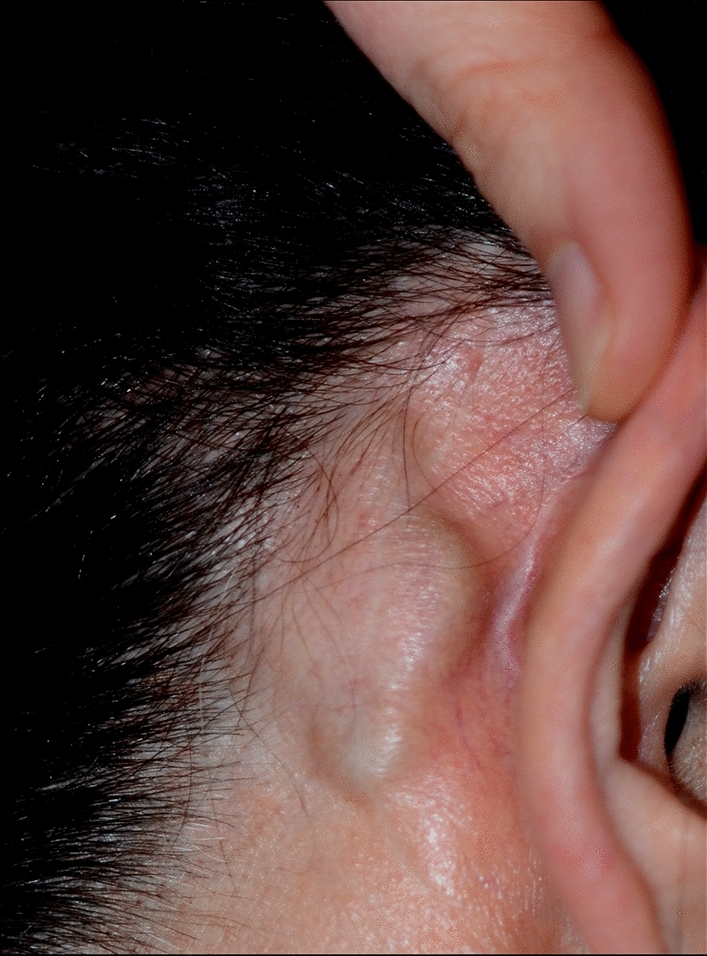
Progressive protrusion of the implant under a preexisting thin retroauricular skin without local signs of irritation or inflammation (patient 14)

One long-term complication was reported, at 4 years after implantation (patient 14). The FMT implant bed needed to be moved more posteriorly towards the retrosigmoidal area to prevent implant protrusion following on progressive outlining of the implant through preexisting thin retroauricular skin (Fig. 5). This procedure was conducted in the same session with a cavity revision and canaloplasty. No further short-, mid-, or long-term minor or major complications were reported.

Because four minor and two major complications related to the implant or the implantation procedure occurred in 32 implantations, the rate of minor complications was 12.5%, and the rate of major complications was 6.2%. Considering that the revision surgery in patient 28 resulted from insufficient thinning of the skin over the receiver coil by the surgeon, the rate of implant-related major complications was 3.1%.

In one case (patient 10) with implant use as CROS in SSD (after neurosurgical removal of vestibular schwannoma), the Bonebridge needed to be explanted approximately 3 years later. After the deterioration of hearing on the contralateral side, the patient no longer experienced any benefit from the CROS.

## Discussion

This analysis of all 32 cases of Bonebridge implantations in children and adults in our department since its market introduction in 2012 yielded insight into the advantages and applicability in various indications and about the limitations and risks of this active BC device.

The audiological results reported here are comparable to those of prior studies [4, 7, 9–15]. We observed a significant improvement in speech recognition (WRS_65_), which is consistent with other studies [4, 10–12, 14, 16–18]. There was even a long-term improvement in WRS_65_ compared to the 1–3-month measurements. This effect was also reported in children by Baumgartner et al. who suggested device acclimation effects and additional fitting procedures as possible factors [16]. Some studies also measured hearing in noise effects using the OLSA or OLKISA [4, 14, 16] or other language tests in noise [11]. These authors all reported a benefit with the use of this transcutaneous BC device of similar magnitude as in this study.

All patients showed stable preoperative and postoperative BC results. Sprinzl et al. [1] calculated the rate of minor adverse events as 5.12% and rate of revision surgery as 0.85%. In a recent meta-analysis, Magele et al. [19] reported a total complications rate of 9.4% (7.7% minor and 1.7% major adverse effects). Compared to the complication rate of other percutaneous devices [3], such as the 23.9% overall complication and 12.1% revision surgery rates reported by Hobson et al., the complication rate of the Bonebridge can be considered lower. We found implant-related complication rates of 12.5% for minor and 3.1% for major complications. It should be considered here that excepting one minor complication (patient 3), all complications occurred in patients with previous ear surgery, which could be a reason for the higher complication rate in our study compared to the complication rates reported in the previously mentioned studies [1, 19]. Furthermore, the complication involving patient 14 occurred 4 years after surgery. Magele et al. reported in their meta-analysis a mean safety follow-up period of 11.7 months, with a range of 3–36 months. Thus, long-term complications arising more than 36 months after surgery, as for patient 14, were not considered [19].

Some of the reported complications were not directly related to the implant. In patient 21 with simultaneous prosthesis anchor implantation, the skin vascularization in the implantation area was reduced after a history of two previous unsuccessful ear reconstruction surgeries, which naturally increased the risk of wound healing complications. Because of insufficient thinning of the skin over the receiver coil at the initial implantation, skin reduction was necessary in patient 28 to provide sufficient attachment force for the sound processor [20]. On the other hand, a protrusion of the implant should be avoided. As noted, in patient 14, whose skin over the implant area showed a preexisting weak and thin structure, a positional change of the FMT became necessary 4 years after the implantation. An implant extrusion complication was reported by Carnevale et al., who resolved that complication with a rotational flap [5].

A basic factor in reducing complications, besides a thorough evaluation of the indication criteria, consists of careful preoperative image-guided planning of the implantation site. In conventional two-dimensional CT scan analysis, to determine the optimal implant position, the experienced surgeon needs to analyze layer by layer in the axial, sagittal, and coronal planes. However, also estimating oblique implant positions requires experience because the three axes of CT scans provide only a perpendicular view, and the transfer of the selected implant position to the intraoperative situation remains challenging. Facing that problem, we developed a 3D planning method [6] that allows free adjustment of the implant model to the skull model and accurate transfer of the position to the intraoperative situation using measurements from landmarks. We applied this method especially in malformations, after multiple ear surgery, and for simultaneous implantation with prosthesis anchors, in which the area of possible FMT implantation was more limited. Here, contact of the implant with the skin-penetrating anchors should be absolutely avoided to reduce infection risk. Furthermore, from an audiological point of view, the distance of the stimulation position to the cochlea seems to be related to hearing thresholds [21], which also can be considered more carefully by means of a 3D model.

Different methods of preoperative planning can rely on software such as “BBfastview” or “3Dslicer” (available for free, http://www1ceit.es/cg/BBFastView and https://www.slicer.org), as well as more complex setups using topographic bone thickness maps [22], navigation [23–26], or 3D-printed template-based methods [27–29].

In our case series, a slight impression (≤ 2 mm) of the dura or sigmoid sinus was necessary in 10 cases. It is difficult to say whether this factor might be relevant to outcomes. Lassaletta et al. [30] found no associations of postoperative pain with dura impression. Vyskocil et al. [18] suggested better sound transmission when the dura was impressed, without reporting complications. On the other hand, headache caused by the new formation of tissue between the implant and the dura was reported in a case that resulted in device explantation [31]. In our study, the impressions were all ≤ 2 mm, whereas Vyskocil et al. [18] reported a range of 2–5 mm. Long-term follow-up data (> 10 years) of dura or sinus impressions are not available yet.

The BCI is another active BC implant that is still in clinical testing [32]. It has smaller dimensions, which might reduce the risk associated with the dimensions of the Bonebridge FMT and facilitate localizing the optimal implant position. Based on a retrospectively analyzed large cohort of children and young adolescents, Rahne et al. described an (hypothetical) optimal form of the FMT as a truncated cone to fit in almost all mastoids [33]. In September 2019, the manufacturer introduced a follow-up model (BCI 602) in Europe, with reduced FMT dimensions, that improved the bone fit in mastoids of children and young adults [34]. However, in certain cases, like very young children, 3D virtual preoperative planning is still recommended [35]. For another, new bone conduction implant it was shown that the average minimum bone thickness was thicker than both the maximum transducer depth of 3 mm and the 2.7 mm bone involvement of the osseointegrating fixation screws. The results indicated implant fit of the new bone conduction implant in all adult patients. Preoperative planning was recommended for children below 9 years old [36].

## Conclusion

We observed a significant audiological benefit after hearing rehabilitation with the Bonebridge. The rate of implant-related adverse events was low. However, given the dimensions of the FMT, preoperative 3D planning is recommended especially in small and hypoplastic mastoids, children, ear malformation, and simultaneous implantation of anchors for ear prosthesis, and after multiple ear surgery.

## Data Availability

All relevant data have been provided in the manuscript. There is no additional or supplementary data.
